# A cross-scanner and cross-tracer deep learning method for the recovery of standard-dose imaging quality from low-dose PET

**DOI:** 10.1007/s00259-021-05644-1

**Published:** 2021-12-24

**Authors:** Song Xue, Rui Guo, Karl Peter Bohn, Jared Matzke, Marco Viscione, Ian Alberts, Hongping Meng, Chenwei Sun, Miao Zhang, Min Zhang, Raphael Sznitman, Georges El Fakhri, Axel Rominger, Biao Li, Kuangyu Shi

**Affiliations:** 1 Department of Nuclear Medicine, University of Bern, Bern, Switzerland; 2 Department of Nuclear Medicine, Ruijin Hospital, Shanghai Jiao Tong University School of Medicine, Shanghai, China; 3 Collaborative Innovation Center for Molecular Imaging of Precision Medicine, Ruijin Center, Shanghai, China; 4 Department of Informatics, Technical University of Munich, Munich, Germany; 5 ARTORG Center, University of Bern, Bern, Switzerland; 6 Gordon Center for Medical Imaging, Massachusetts General Hospital, Harvard Medical School, Boston, MA USA

**Keywords:** Deep learning, Low-dose, PET, Recovery, Cross-scanner, Cross-tracer

## Abstract

**Purpose:**

A critical bottleneck for the credibility of artificial intelligence (AI) is replicating the results in the diversity of clinical practice. We aimed to develop an AI that can be independently applied to recover high-quality imaging from low-dose scans on different scanners and tracers.

**Methods:**

Brain [^18^F]FDG PET imaging of 237 patients scanned with one scanner was used for the development of AI technology. The developed algorithm was then tested on [^18^F]FDG PET images of 45 patients scanned with three different scanners, [^18^F]FET PET images of 18 patients scanned with two different scanners, as well as [^18^F]Florbetapir images of 10 patients. A conditional generative adversarial network (GAN) was customized for cross-scanner and cross-tracer optimization. Three nuclear medicine physicians independently assessed the utility of the results in a clinical setting.

**Results:**

The improvement achieved by AI recovery significantly correlated with the baseline image quality indicated by structural similarity index measurement (SSIM) (*r* = −0.71, *p* < 0.05) and normalized dose acquisition (*r* = −0.60, *p* < 0.05). Our cross-scanner and cross-tracer AI methodology showed utility based on both physical and clinical image assessment (*p* < 0.05).

**Conclusion:**

The deep learning development for extensible application on unknown scanners and tracers may improve the trustworthiness and clinical acceptability of AI-based dose reduction.

**Supplementary Information:**

The online version contains supplementary material available at 10.1007/s00259-021-05644-1.

## Introduction

Positron emission tomography (PET) is one of the main imaging modalities in clinical routine procedures of oncology [1, 2], neurology [3], and cardiology [4]. One of the critical bottlenecks for the wide application of PET is the ionizing radiation dose [5]. Although the general principle of as low as reasonably achievable (ALARA) [5] is followed in clinical practice, patients are typically exposed to more than 4 mSv of equivalent dose [6]. In general, the imaging quality of PET is directly influenced by the activity of the injected tracer and the consequent radiation dose. A reduction of the radiation dose in PET protocols however leads to the degradation of imaging quality.

The technical advancement of PET scanners in recent decades has steadily reduced the radiation burden while preserving the imaging quality [7]. Breakthroughs have been made in signal measurement and imaging generation, including developments on scintillator crystals, photodetectors, acquisition electronics, and image reconstruction techniques [8]. Modern commercial PET scanners have introduced time-of-flight (TOF) techniques at a higher level of coincidence time resolution, which largely improved image quality [9–11]. Analog scanners are still commercially available but are increasingly being replaced by solid-state solutions. The transition of all major commercial vendors to silicon photomultiplier (SiPM)-based (digital) scanners has enabled a much-improved TOF resolution [12–15], as well as higher sensitivity, which increased measurement efficiency [16–18] and might afford radiation dose reductions of more than 40% [19–21]. The recent innovation of total-body PET technology further improves the sensitivity of PET and may allow for further reductions of radiation exposure associated with PET imaging [22–25]. However, such high-end scanners are only available in a small number of centers.

By contrast, computational techniques provide an alternative, cost-effective solutions to improve image quality for low-dose PET imaging. Denoising methods such as nonlocal means [26] or multi-scale curvelet and wavelet analysis [27] were developed to reduce the noise in low-dose PET images. Data-driven methods have been employed to synthesize high-quality standard-dose PET images from low-dose measurements using machine learning, such as random-forest-based regression [28], mapping-based sparse representation [29], semi-supervised tripled dictionary learning [30], multilevel canonical correlation analysis framework [31], and so on. However, these small patch-based learning estimations may result in over-smoothed images lacking texture information that limit the quantification of small structures in synthesized PET images. Recently developed deep learning techniques have been shown to better predict textural information in radiological images. Xiang et al. [32] proposed a concatenated end-to-end convolutional neural network (CNN) to estimate full-dose PET images, which effectively utilize the structural information from input data.

One challenge in deep learning is defining an analytical error function that enables an image quality perception comparable to human perception. GAN [33] is a special type of neural network model consisting of two units, with the generator unit synthesizing candidates while the discriminator unit attempts to decipher whether the candidate’s images are synthetic or real. The development of GAN has strengthened the capability of neural networks in this regard, allowing them to capture complex probability distributions. Wang et al. employed the adversarial training scheme to recover full-dose PET images from low-dose PET using a conditional GANs model [34] and further improved the performance by incorporating MRI images that provide extra anatomical information [35].

However, the translation of this technology to a clinical setting is not straightforward. PET imaging is characterized by the variability of instrumentation and imaging protocols [36, 37], such as geometric configuration, detector capability (e.g., TOF [38], depth-of-interaction (DOI) [39]), data correction, and system calibration. Furthermore, PET imaging is also strongly influenced by the variability of injected radiopharmaceuticals. Even in different tracers using the same radioisotope, the signal texture may be different due to other different molecules of the tracers. This issue may be especially important for the development of new tracers, where PET datasets from new or uncommonly used tracers may not be adequately available. Moreover, the trustworthiness of AI has been rigorously questioned over the last decade, for its reproducibility and stability when applied to external datasets.

Therefore, our goal was to develop and optimize a deep learning method for the recovery of standard-dose imaging quality from low-dose PET in a versatile clinical setting, including different imaging instrumentations and radiopharmaceuticals.

## Materials and methods

### Patient cohorts

The study was conducted in accordance with the requirements of the respective local ethics committees in Switzerland and China. Seven cohorts with 310 subjects were retrospectively included in this study (Table 1). For the Chinese cohorts, we selected 255 subjects who referred to [^18^F]FDG PET for various non-neurological/psychiatric purposes and that were considered neurologically healthy on PET imaging between April and December 2019. We also selected 10 patients who underwent [^18^F]Florbetapir PET for suspected neurodegenerative disease between April and August 2021. For the Swiss cohorts, we selected 27 patients who underwent [^18^F]FDG PET for suspected neurodegenerative disease and 18 patients who underwent [^18^F]FET PET for suspected brain tumors between February and November 2019.

**Table 1:** Information on patients’ demographics and diagnosis

Diagnosis	Development group—healthy	Test group—neurodegeneration			Test group—brain tumor		
**Scanner**	GE Discovery MI	GE Discovery MI	Siemens Biograph mCT	Siemens Biograph Vision	GE Discovery MI	Siemens Biograph mCT	Siemens Biograph Vision
**Tracer**	[^18^F]FDG	[^18^F]Florbetapir	[^18^F]FDG	[^18^F]FDG	[^18^F]FDG	[^18^F]FET	[^18^F]FET
**Location**	China	China	Switzerland	Switzerland	China	Switzerland	Switzerland
**Scan results (number of patients)**	Control group (237)	Scan negative for Alzheimer (1)	Normal scan (10)	Normal scan (4)	Scan negative for brain tumor (8)	Scan negative for brain tumor (6)	Scan negative for brain tumor (4)
		Scan positive for Alzheimer (9)	Neurodegeneration (10)	Neurodegeneration (3)	Scan positive for brain tumor (10)	Scan positive for brain tumor (4)	Scan positive for brain tumor (4)
**Gender (male/female)**	127/110	5/5	14/6	4/3	12/6	7/3	5/3
**Age (year)**	56.4 ± 14.0	76.5 ± 6.1	64.6 ± 14.3	63.0 ± 22.8	60.9 ± 9.3	55.7 ± 14.8	57.3 ± 9.5
**Weight (kg)**	63.5 ± 13.2	65.3 ± 12.4	73.8 ± 10.7	73.9 ± 16.6	64.2 ± 11.9	81.3 ± 19.8	77.0 ± 15.6
**Total dose (MBq)**	353.5 ± 6.6	325.5 ± 22.0	249.7 ± 6.3	240.6 ± 3.1	330.3 ± 76.3	249.6 ± 15.3	252.8 ± 11.4
**Post-injection uptake time (min)**	89.7 ± 87.2	47.9 ± 11.7	36.6 ± 6.9	33.6 ± 3.0	69.6 ± 24.0	33.0 ± 5.1	35.4 ± 4.8
**Standard full dose acquisition time (min)**	5	15	15	15	5	20	20
**Dose reduction factor**	2,4,10,20	2,4,10,20,50,100	2,4,10,20,50,100	2,4,10,20,50,100	2,4,10,20	2,4,10,20,50,100	2,4,10,20,50,100

The subjects were scanned on 3 different PET scanners (GE Discovery MI, Siemens Biograph mCT, Siemens Biograph Vision) with 3 different tracers ([^18^F]FDG, [^18^F]Florbetapir, and [^18^F]FET). The first cohort consists of 237 subjects considered neurologically healthy referred to [^18^F]FDG PET on DMI (GE, Discovery MI), which was employed for the development of our deep learning methods. The second cohort consists of 10 patients for suspected neurodegenerative disease, who underwent [^18^F]Florbetapir PET were scanned on DMI. The third and fourth cohorts with suspected neurodegeneration were scanned on a mCT (Siemens, Biograph mCT) (*n* = 20) and Vision (Siemens, Biograph Vision) (*n* = 7) with [^18^F]FDG. The fifth cohort contained 18 subjects with suspected brain tumors in the brain who underwent [^18^F]FDG PET on DMI. The last two cohorts with suspected brain tumors were acquired on mCT (*n* = 10) and Vision (*n* = 8) with [^18^F]FET PET.

### Imaging protocols

All data was acquired in list mode allowing for rebinding of data to simulate different acquisition times. PET data were reconstructed using OSEM (ordered subset expectation maximization). More detailed information concerning scanner properties and reconstruction parameters can be found in Supplementary Table S1. Each simulated low-dose PET with a certain dose reduction factor (DRF) was reconstructed from the counts of a time window resampled at the middle of the acquisition with correspondingly reduced time. For example, the full-dose PET images from the DMI are reconstructed with 5-min raw data, while the simulated low-dose PET with DRF = 2 is reconstructed with 2.5-min (from the 75th second to the 225th second) resampled raw data but with the same reconstruction parameters and post-processing procedure, ensuring that both images have a comparable spatial resolution.

### Deep neural network setup

**Fig. 1:**
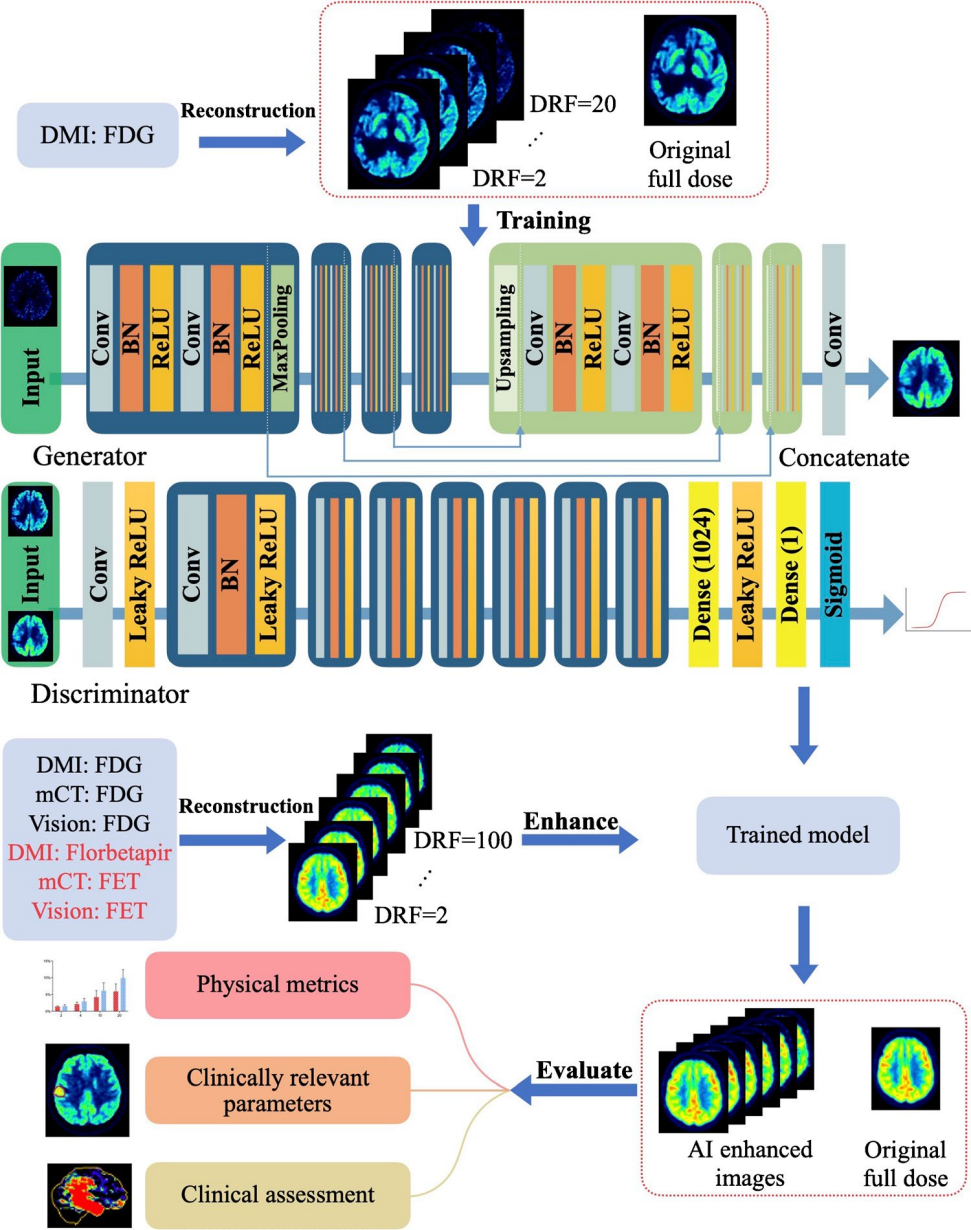
An illustration of our proposed method, including data collection, neural network training, image enhancement, and final evaluation. The model was trained by mixing the image pairs of all DRF up to 20 from DMI (GE, Discovery MI), and later tested on datasets from different scanners and tracers with DRF up to 100. We evaluated the results with physical metrics, clinical-relevant parameters, and with clinical assessment by two independent nuclear medicine physicians

Our network was developed based on the conditional GANs (c-GANs) [33, 34] architecture, which consists of a generator network to synthesize the full-dose images from low-dose measurements and a discriminator to distinguish between the synthesized full-dose image and the real input. As shown in Figure 1, we specifically customized our model for cross-scanner and cross-tracer application including a U-net like architecture featuring skip connection (referred to as “Concatenate” in Figure 1) [40], batch normalization (BN) [41], a modified objective function with both conventional content loss [33] and also voxel-wise loss. Techniques like skip connection and BN allow the network architecture to be much deeper, which endows the network with a better capability of generalization. Customized loss function helps to preserve complex image details. The model was trained by mixing the image pairs of all DRF up to 20 from DMI and later tested on datasets from different scanners and tracers with DRF up to 100. More information on the network design and training procedure is attached in the corresponding part of the Supplementary material.

### Evaluation based on physical metrics

To evaluate the quality of the enhanced images on all test datasets, we calculated and compared the physical metrics including the normalized root mean squared error (NRMSE) which measures the overall pixel-wise intensity deviation, peak signal-to-noise ratio (PSNR) as well as structural similarity index measurement (SSIM) that reflects perceived image quality [42]. Differences between the AI-enhanced and non-AI-enhanced groups for NRMSE were assessed for statistical significance by means of the paired two-tailed *t*-test. Furthermore, to examine the level of difference of AI enhancement in a cross-scanner and cross-tracer setting, an unpaired two-tailed *t*-test was performed for NRMSE improvement (percentage error calculated between AI-enhanced and non-AI-enhanced groups) on results from all three scanners and both included tracers. A *p*-value lower than 0.05 was considered statistically significant.

### Clinical assessment for cross-scanner application

For the cross-scanner assessment, the neurodegeneration cohorts imaged with [^18^F]FDG (scanned with mCT *n* = 20 and Vision *n* = 7) were assessed with NEUROSTAT/3D-SSP [43] according to a standardized procedure used in everyday clinical practice, comparing each patient’s images with an age-matched healthy collective.

In a first step, the 3D-SSP results as well as complete axial images (full-dose, AI-enhanced, and non-AI-enhanced low-dose images from DRF 2 to 100) of each patient were directly visually compared with each other by two board-certified nuclear medicine physicians (A.R. and K.P.B.). Subsequently, the physicians determined at which DRF the AI-enhanced images started preserving a better diagnostic value in comparison with non-AI-enhanced images and thus came closer to full-dose images. In a second step, the two nuclear medicine physicians independently assessed three subsets of the images of the neurodegeneration cohorts (full-dose, DRF = 50 with and without AI enhancement) as explained in the following passage. The DRF = 50 subset was chosen based on the results of the first step. The physicians were blinded regarding the source of the image (e.g., full-dose or DRF image) as well as patient clinical information.

The results from the 3D-SSP analysis were rated regarding the visual hypometabolism compared to healthy controls in four regions (frontal, parietal, temporal lobe, and PCC), for each hemisphere on a four-point scale (0 = no hypometabolism, 1 = little hypometabolism, 2 = medium hypometabolism, 3 = strong hypometabolism). The results of the rating were also simplified to a binary scale (0 = no or little hypometabolism, 1 = medium or strong hypometabolism). The four-point scale and binary results of the rating were compared between the three subsets by the Friedman test (*p* < 0.05) for significant differences using SPSS Version 25.0. In case of significant differences on the Friedman test, additional post hoc tests using Wilcoxon signed-rank test with Bonferroni adjustment were performed, with *p* < 0.017 considered significant.

### Clinical assessment for cross-tracer application

For the cross-tracer assessment, [^18^F]Florbetapir standardized uptake value ratio (SUVR) maps were generated using the cerebellum gray matter as reference regions, for the purpose of visual assessment [44] by a nuclear medicine physician. As for the brain tumor cohorts (imaged with [^18^F]FDG and [^18^F]FET), we measured clinical imaging parameters such as SUVmean, SUVmax, as well as the most relevant radiomics features [45] described in literature within [46–55]. The lesions were delineated manually and reviewed by a board-certified nuclear medicine physician. The accuracy of the clinical imaging parameters and radiomics features of the lesions were calculated in reference to full-dose images (percentage error). The results of the AI-enhanced and non-AI-enhanced groups were compared at all DRFs. More detailed information regarding feature selection and the analysis procedure can be found in the corresponding part of the Supplementary material.

## Results

### Physical metrics evaluation for cross-scanner application

**Fig. 2:**
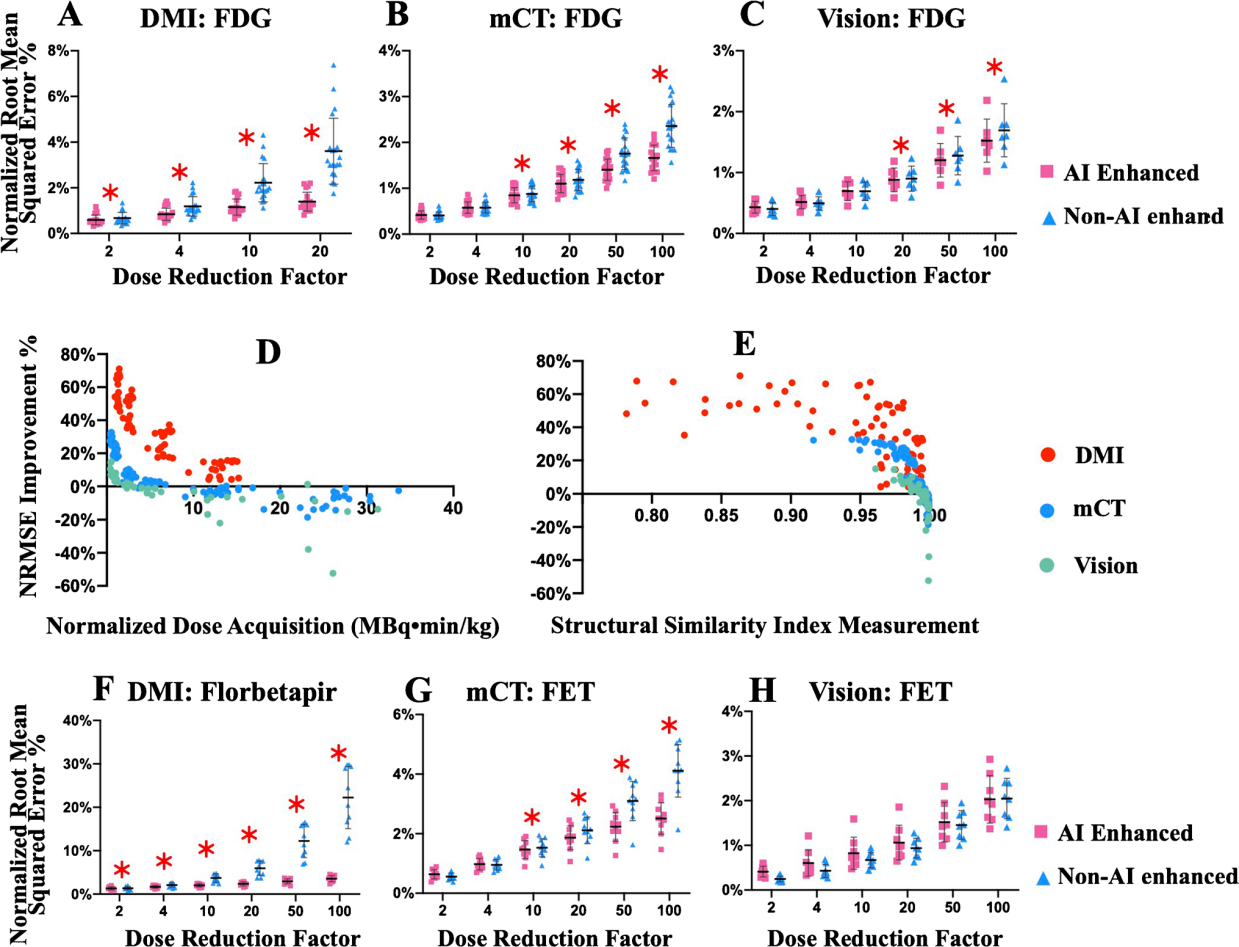
Improvement with the help of the developed AI enhancement in terms of NRMSE in a cross-scanner and cross-tracer setting. ***A***–***C*** Comparison of NRMSE between images with and without AI enhancement at different dose reduction factors (DRFs) for the [^*18*^F]FDG data from DMI (***A*** GE, Discovery MI), mCT (***B*** Siemens, Biograph mCT) and Vision (***C*** Siemens, Biograph Vision), respectively. The asterisk denotes the DRF, where the AI-enhanced group showed a significant advantage compared to the non-AI-enhanced group based on *t*-test results (*p* < 0.05). ***D***–***E*** Improvement of NRMSE with regard to the normalized dose acquisition correcting for acquisition time and patient weight (***D***) and SSIM (structural similarity index measurement) (***E***). ***F***–***H*** Comparison of NRMSE between images with and without AI enhancement at different DRFs for the [^*18*^F]Florbetapir data from DMI (***A*** GE, Discovery MI), [^*18*^F]FET data from mCT (***B*** Siemens, Biograph mCT), and Vision (***C*** Siemens, Biograph Vision), respectively

The customized c-GAN trained on [^18^F]FDG images from DMI was tested on [^18^F]FDG images on three different scanners. The results for NRMSE on [^18^F]FDG imaging are shown in Figure 2. Figure 2A, B, and C showed that NRMSE improvement using AI tended to increase with increasing DRF on all three scanners. Compared to non-AI-enhanced group, the AI-enhanced group achieved statistically significant advantage for the paired *t*-test on DMI from DRF = 2 (*p* = 1.8E−6), on mCT from DRF = 10 (*p* = 4.5E−5), and on Vision from DRF = 20 (*p* = 0.03). Additional results of PSNR and SSIM on [^18^F]FDG imaging on the three different scanners showed the same tendency as the NRMSE results (Supplementary Figure S2).

Figure 2D and E illustrated the improvement by AI enhancement referring to baseline image quality. The baseline image quality (*x*-axis) was represented by the normalized dose acquisition (D), which is the injecting dose corrected for acquisition time and patient weight, and SSIM (E) of the non-AI-enhanced images. The NRMSE improvement (*y*-axis) on low-dose images by using AI enhancement significantly negatively correlated with the baseline image quality (normalized dose acquisition: *r* = −0.60, *p* = 3.6E−24 and SSIM: *r* = −0.71, *p* = 1.1E−37).

Figure 2A–E overall suggested that the benefits of AI increase with decreasing image quality and the image quality degradation of mCT and Vision was less affected by the dose reduction and was milder compared to DMI. The unpaired *t*-test results illustrated that the application of AI on different scanners achieved comparable results, although not as good as the trained scanner (DMI). For example, the NRMSE improvement on mCT at DRF = 100 achieved the same level as in the case of DRF = 4 on DMI (*p* = 0.12). The level of improvement on Vision at DRF = 100 achieved the same level as in the case of DRF = 2 on DMI (*p* = 0.63).

**Fig. 3:**
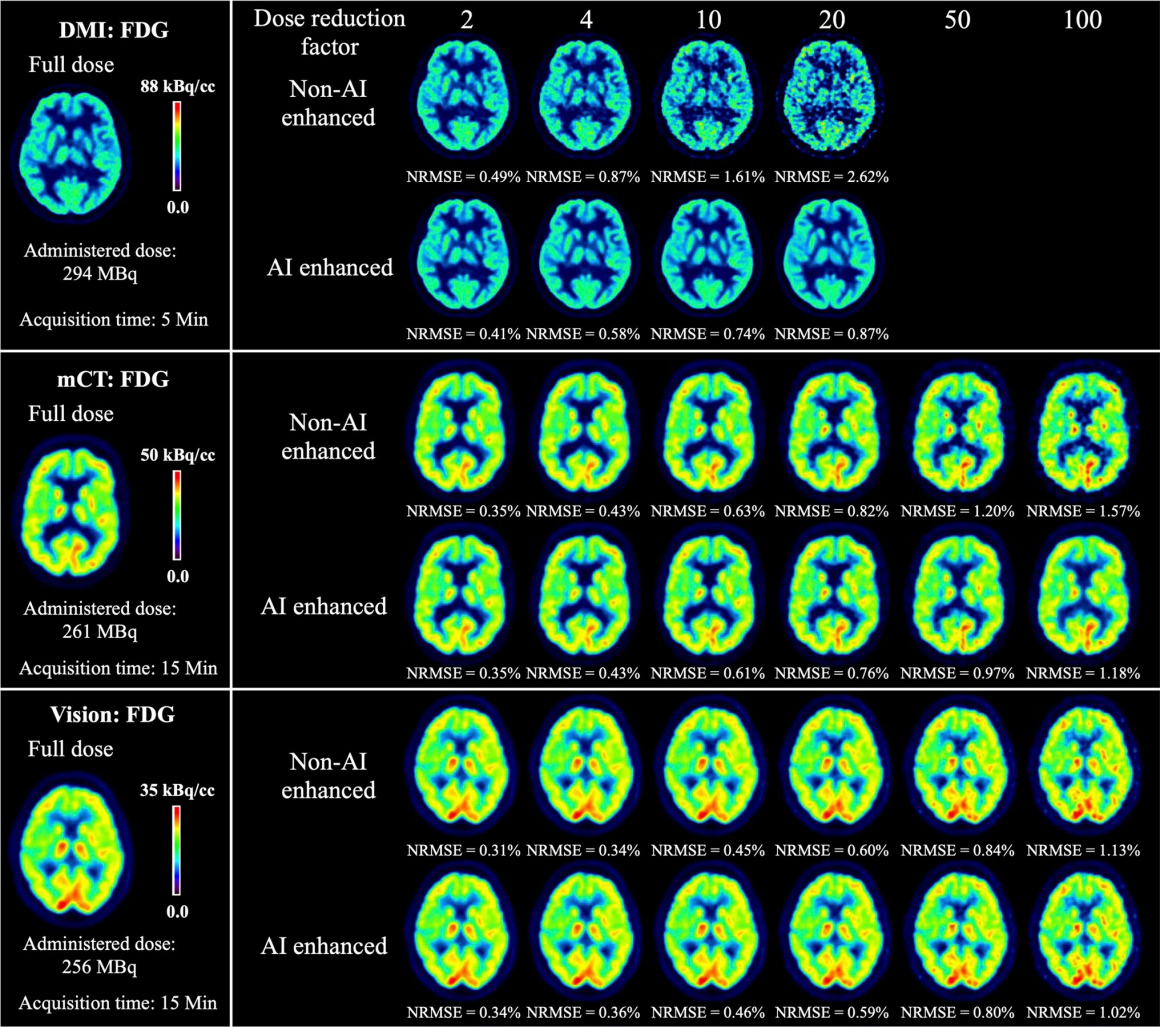
Example of test results of [^18^F]FDG imaging from DMI (GE Discovery MI), mCT (Siemens, Biograph mCT), and Vision (Siemens, Biograph Vision)

The aforementioned points were also confirmed by the visual reading (Figure 3), namely that our model was able to enhance image quality on all three scanners, especially at high DRF. AI enhancement achieved overall good performance on DMI. As for the mCT data, AI enhancement started to show its advantages from DRF = 50, with the non-AI-enhanced images still maintaining good image quality under DRF = 50. The level of improvement on Vision was not as evident as on mCT.

### Physical metrics evaluation for cross-tracer application

The same trained c-GAN was tested on cross-tracer data from three different scanners. The results for NRMSE are shown in Figure 2F–H. Compared to the non-AI-enhanced group, the AI-enhanced group achieved statistically significant advantage for the paired *t*-test on [^18^F]Florbetapir (DMI) from DRF = 2 (*p* = 0.03), on [^18^F]FET (mCT) from DRF = 10 (*p* = 0.001), no significant advantage observed on [^18^F]FET (Vision). Furthermore, the unpaired *t*-test results illustrated that there were no statistically significant differences between the application of AI to a different tracer with the same scanner. For example, the NRMSE improvement on [^18^F]Florbetapir achieved almost the same level as [^18^F]FDG (*p* = 0.6, DRF = 10; *p* = 0.8, DRF = 20). Additional results of PSNR and SSIM on both tracers showed the same tendency as the NRMSE results (Supplementary Figure S2).

**Fig. 4:**
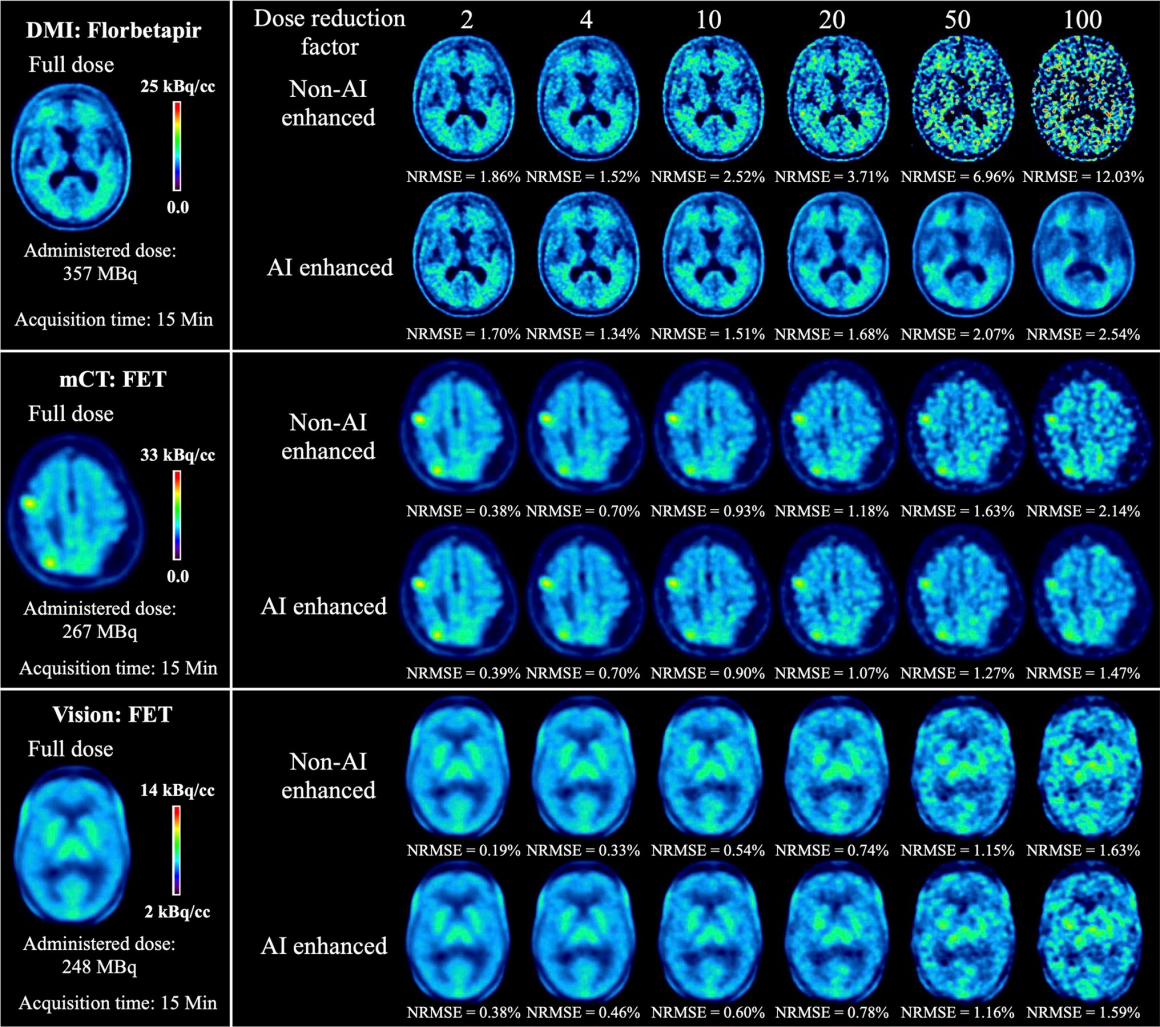
Example of test results from DMI (GE Discovery MI) with [^18^F]Florbetapir, mCT (Siemens, Biograph mCT), and Vision (Siemens, Biograph Vision) with [^18^F]FET

The aforementioned points were also confirmed by the visual reading (Figure 4), namely that our model was able to enhance image quality for both [^18^F]Florbetapir and [^18^F]FET, especially at high DRF. AI enhancement achieved overall good performance on [^18^F]Florbetapir. As for [^18^F]FET, AI enhancement started to show its advantages from DRF = 50 on mCT. The level of improvement on Vision was not as evident as on mCT.

### Clinical assessment for cross-scanner application

**Fig. 5:**
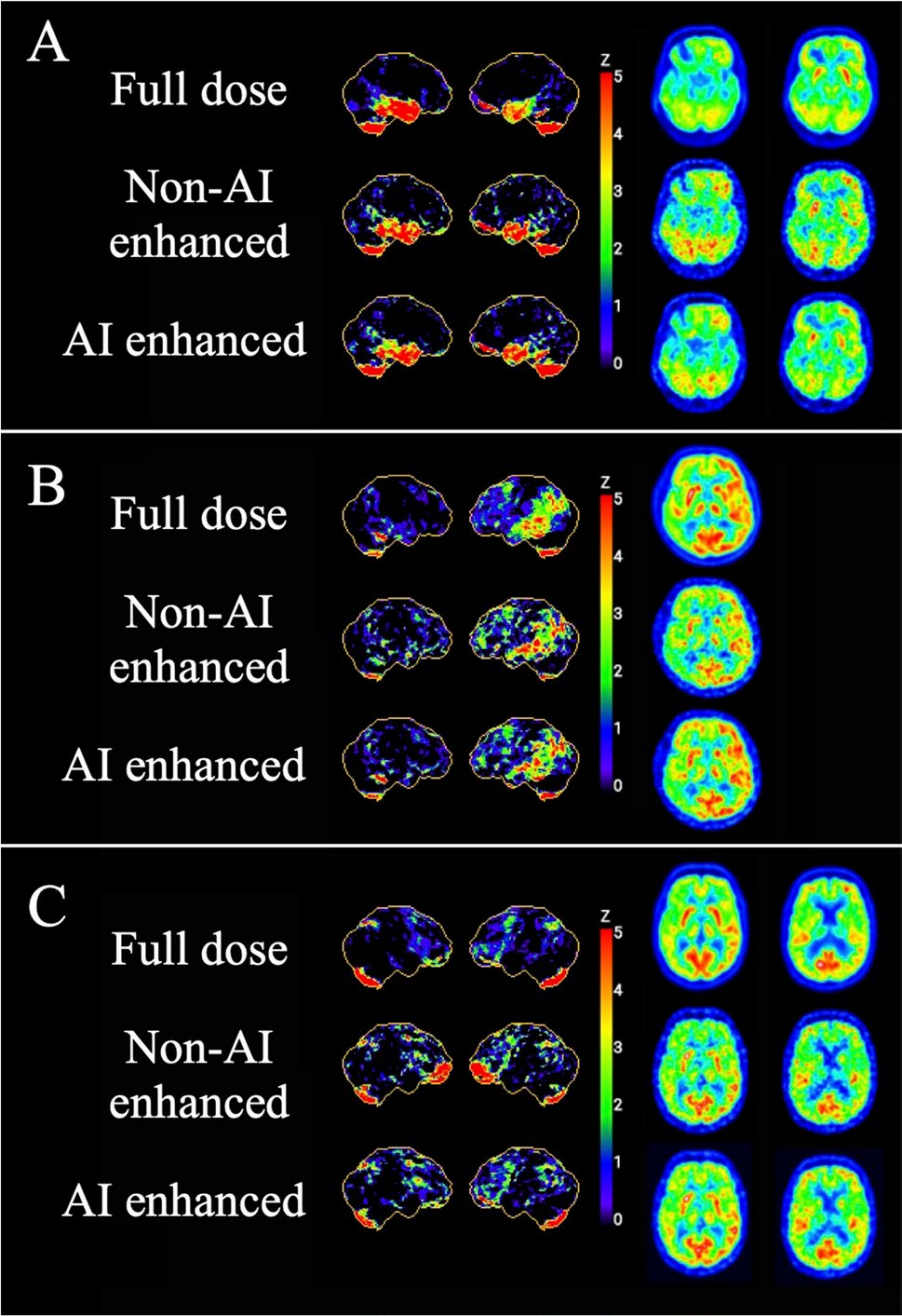
Example [^18^F]FDG images of 3D-SSP results with global cortex as reference region and corresponding axial slides for full-dose, non-AI-enhanced, and AI-enhanced images at DRF = 50 from mCT (Siemens, Biograph mCT). **A** Male, 59 years old, referred with Alzheimer’s disease and additional clinical signs of a frontal lobe disorder. **B** Male, 68 years old, referred with progressive word finding and memory difficulties, cerebrospinal fluid with pathological amyloid findings in favor of Alzheimer’s disease. Clinically inconclusive findings (DDx Alzheimer’s dementia, frontotemporal dementia, vascular dementia, Lewy body dementia). **C** Male, 71 years old, referred with a suspicion of progressive supranuclear palsy

The comparison of the 3D-SSP data and the axial images of the neurodegeneration data for all available DRF showed an advantage of AI enhancement starting at DRF = 50 in most cases. This was mainly due to mCT data, which makes up the biggest part of the neurodegeneration group. For DRF = 50, non-AI-enhanced images tended to be more blurred and to overestimate the extent of pathology. For example, in Figure 5A, all 3D-SSP images showed a fairly stable pattern of predominantly temporal bilateral hypometabolism, with a slight tendency of the non-AI-enhanced images to be more blurred. The corresponding axial images showed the disadvantages of the non-AI-enhanced images clearer, as they were overall more blurred, and as the areas of temporal hypometabolism were harder to separate from the adjacent non-affected areas, as well as basal ganglia being less demarcated. The increased tendency of non-enhanced images compared to AI-enhanced images to overestimate the extent of pathology can be seen in the frontal lobes in Figure 5B.

In some cases, the 3D-SSP results of the non-AI-enhanced images even showed strong incorrect/artificial hypometabolism of some regions, which was not visible in the 3D-SSP results of the full-dose images. This is demonstrated by Figure 5C, where non-AI-enhanced images showed bilateral frontal hypometabolism, which could not be seen on full-dose or AI-enhanced images. This erroneous frontal hypometabolism on non-AI-enhanced images was not visible for images under DRF = 50. More examples can be found in Supplementary Figure S6.

In contrast, the effect of AI enhancement was not as evident on data from Vision, being the scanner with the overall best imaging quality (Supplementary Figure S7).

On an additional inspection, AI enhancement performed best on data from DMI, with the advantage of AI being particularly evident in the case of high DRF or poor image quality. An exemplary case is shown in Supplementary Figure S8.

The rating of the 3D-SSP data also showed an overall advantage of AI enhancement. The Friedman test showed significant differences (*p* < 0.05) between the three assessed groups for rater 1 on the four-point scale (*p* = 0.017, *χ*^2^ 8.133) and the binary scale (*p* = 0.002, *χ*^2^ 12.133), whereas there were no significant differences for rater 2 (four-point scale *p* = 0.551, binary scale *p* = 0.472). For rater 1, the following post hoc test showed significant differences between the full-dose and the DRF = 50 non-AI-enhanced groups (four-point scale *p* = 0.005, binary scale *p* = 0.013), and partly between the DRF = 50 non-AI-enhanced and AI-enhanced groups (four-point scale *p* = 0.133, binary scale *p* = 0.004). No significant differences were found between the full-dose and DRF = 50 AI-enhanced groups.

### Clinical assessment for cross-tracer application

**Fig. 6:**
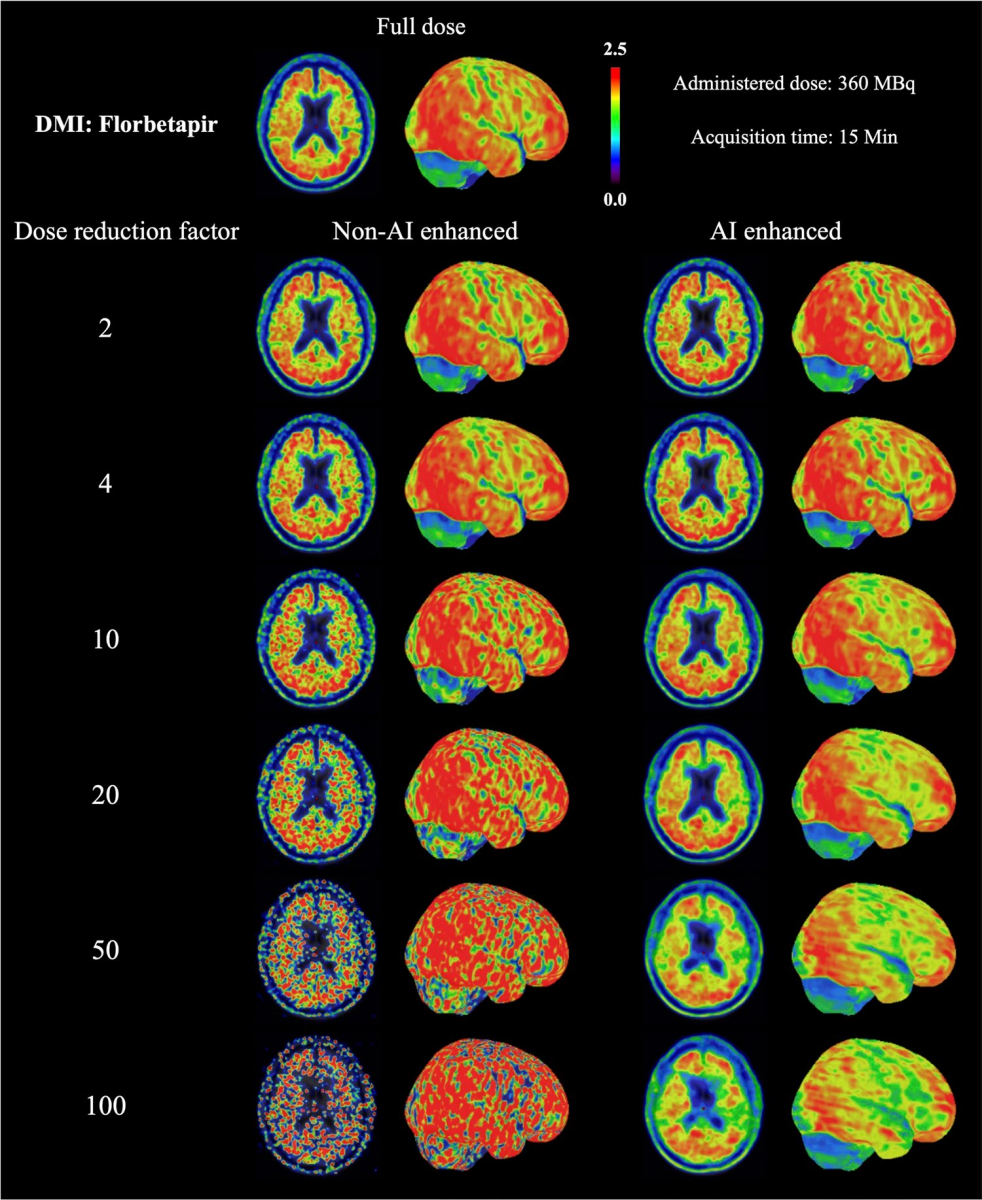
Example [^18^F]Florbetapir images of Alzheimer’s disease on DMI (GE, Discovery MI) for full-dose and different DRFs with or without AI enhancement. Results showed that the enhanced images preserved an overall better image quality and that the improvement tended to increase with higher DRF, but some inconsistencies were observed compared to the full-dose image

Results of [^18^F]Florbetapir dataset showed an overall advantage of AI enhancement, especially starting from DRF 10. The most noticeable improvement in image quality was observed with DRF 100, but some inconsistencies were observed compared to the full-dose images (Figure 6).

**Fig. 7:**
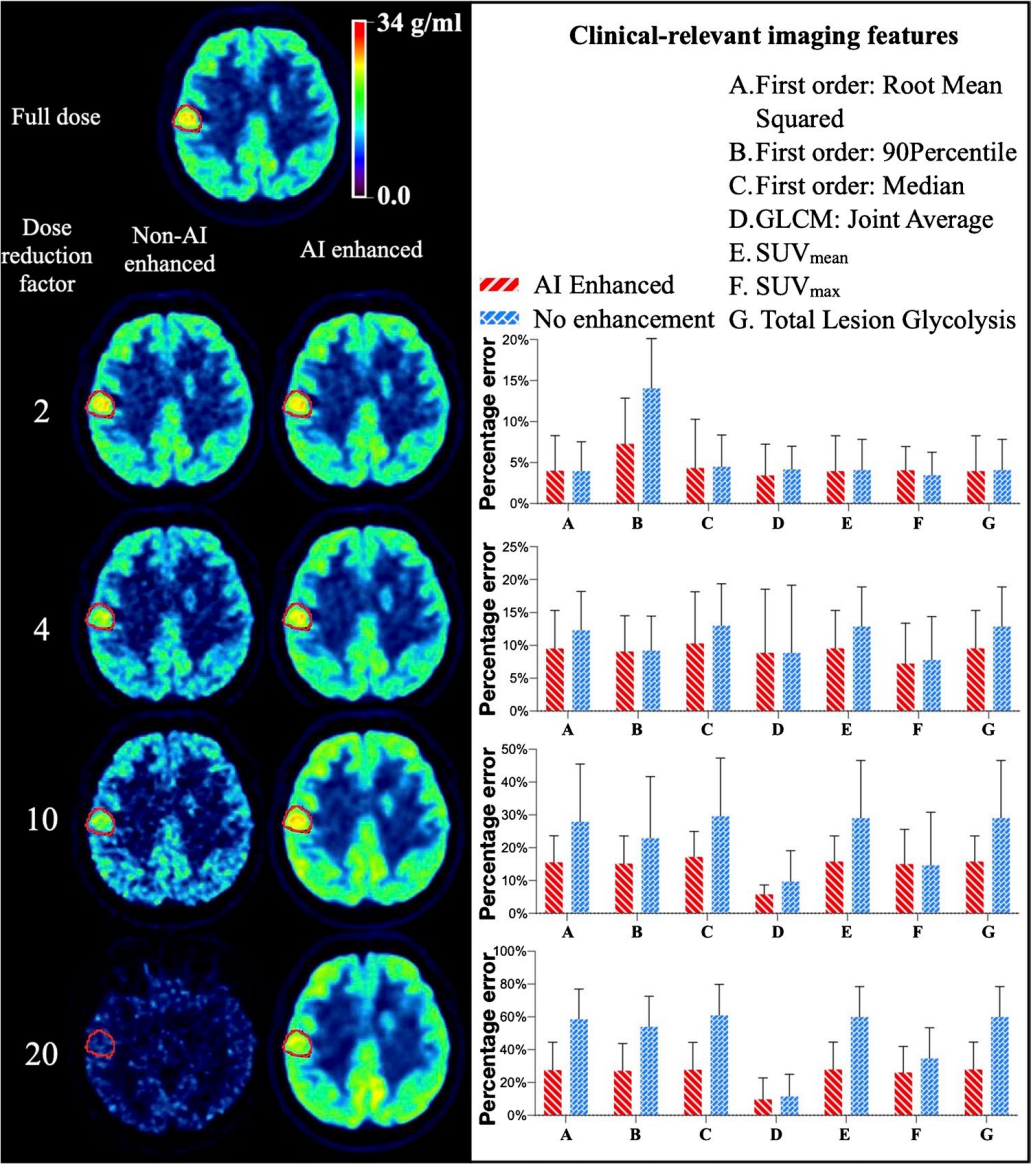
Clinical features analysis on DMI (GE, Discovery MI) with [^18^F]FDG. Results show that the enhanced images preserve an overall better quality in terms of those features and that the improvement compared to non-enhanced data tends to increase with higher DRF

Regarding the brain tumor dataset, results of [^18^F]FDG imaging from DMI suggested that the AI-enhanced images overall preserved an improved quality in terms of the selected features and the improvement tended to increase with higher DRF (Figure 7). Yet, none of the clinical features of the [^18^F]FET images benefited from the enhancement (Supplementary Figure S5). Additional results of lesion segmentation and analysis can be found in Supplementary Figures S4 and 5.

## Discussions

A critical concern when using machine learning is its reproducibility and extensibility to unknown complexity in real application [56]. Methods optimized in one cohort have been reported to have limited performance in other cohorts or other applications [57]. Despite the demonstrable potential of AI for PET dose reduction, the main challenge for its clinical translation for routine clinical use remains its ability to take the large complexity involved in molecular imaging into account, such as the variety of tracers, scanners, imaging protocols, reconstruction settings, metabolic dynamics, and so on [36, 37]. The strength of this study lies in its trustworthy design. The model, trained with data from one center was applied to data from different scanners, diseases, and tracers in another center. Our results demonstrated that the customized deep learning was able to synthesize images comparable to full-dose PET images from low-dose PET images with certain restrictions. The improved capability of cross-scanner and cross-tracer application can enhance the translational credibility of the AI methods in nuclear medicine, considering the diversity and rapid growth of new instruments and radiopharmaceuticals. Our study attempts to explore the translational potential of deep learning for low-dose PET protocols in-depth and for moving a step toward clinical practice.

We included both digital and analog scanners for variability. The digital PET scanners were equipped with SiPM that enables higher efficiency and better TOF measurements, compared to conventional analog PET scanners [58, 59], which is a major source of variability of input image quality. Our results indicated that although our model was developed based on a digital scanner (DMI), AI tends to be more helpful when recovering from low-dose PET on an analog scanner (mCT). Considering the overall better properties of the digital scanner, e.g., producing images with higher spatial resolution and less noise or artifacts, less room for AI improvements seems to be left. Acquisition protocols including aspects of injected dose or acquisition time may also contribute to variability. As shown in Table 1 and Supplementary Table S2, the two included centers follow different protocols each with respect to different local conditions. As shown in Figure 2D, owing to longer acquisition time, the image quality degradation of mCT and Vision was less affected by the dose reduction compared to DMI, especially in the case of the SiPM-based digital scanner (Vision) as seen in Figure 2E. Therefore, we additionally obtained DRF = 50, 100 data from both Siemens scanners (mCT and Vision) to make the data more comparable. Additionally, image reconstruction was performed using manufacturer-provided software with recommended parameters, which differ in several aspects such as iterations and subsets when performing iterative reconstruction with OSEM [60]. Algorithms for physical corrections including attenuation and scatter corrections also vary between scanners. The reconstruction procedures deliberately followed the vendors’ recommendations and were in line with normal clinical settings, in order to fairly assess the robustness of the proposed model in handling routine applications. Despite all the aforementioned variabilities in the cross-scanner application setting, the results demonstrated that our customized c-GAN was able to achieve a comparable level of enhancement regarding image quality.

The clinical assessment overall showed AI to be advantageous when applied to low-dose PET images. Although the clinical and physical evaluations were carried out independently, the results were consistent with each other. Accordingly, the clinical evaluation also showed that the positive effect of AI becomes greater with decreasing image quality as shown in Figures 3, 4, 5, and 6. As the clinical evaluation had a focus on DRF = 50 on the cross-scanner setting, the benefit of AI should be evaluated in a clinical setting with even higher DRFs. Nevertheless, it also remains unclear how AI enhancements will perform in a real clinical setting, in which the raters have further clinical information that they can use to interpret the images and come to a conclusion/diagnosis. Therefore, it needs further assessment in a routine setting and within larger cohorts. Furthermore, it should be possible to significantly reduce the dose without a relevant impact on clinical assessment results or image quality, even without the use of AI, e.g., up to DRF = 20 on mCT data. However, we should also be aware of cases like the one in Figure 5C where the 3D-SSP results of the non-AI-enhanced images showed strong incorrect/artificial hypometabolism for DRF = 50 which might lead to a false diagnosis in clinical routine. In such a situation, information might have been affected during the 3D-SSP processing pipeline. Since the corresponding axial slices showed the same findings independently of the 3D-SSP data, we can state that this was not the case. In summary, the clinical evaluations showed that AI is beneficial, especially in the cross-scanner application of AI enhancement on mCT data.

We employed imaging with the same radioisotope but different molecules for the tracer. AI overall performed well on [^18^F]Florbetapir imaging of Alzheimer’s disease, since it was acquired from the same scanner as the training dataset (DMI). The improvement became more evident starting from DRF 10, while inevitably producing some artifacts at a higher reduction rate (100), which must be treated with caution when diagnosing. This fact may also be related to the highest DRF included in our training is only 20. We observed that AI enhancement led to an increase in NRMSE for [^18^F]FET imaging obtained from Vision (Figure 2H), which was most pronounced at low DRF. This can be explained that our current AI training may have limited performance when dealing with complicated situations, i.e., cross-scanner and cross-tracer at the same time. The large variability imposed in both cross-tracer and cross-scanner can place too much burden on the AI model trained with limited complexity. Future work of incorporating diverse training data may overcome the limitation and further improve the performance of AI.

Overall, some limitations of AI application and potential risks need to be considered. There might be some hidden problems associated with GAN technology in image synthesis such as feature hallucination, where GANs may add or remove image features since the source and target domain distributions are paired data [61]. It is therefore important to recruit domain experts to further evaluate the resulting images, considering that physical indicators often fall into this trap. Another limitation of this study is the inherent bias in the limited datasets and the inclusion of additional subjects may further improve the generalizability and robustness of the developed model. Additionally, the low-dose images are simulated by reconstructions with shorter acquisition time and do not originate from patients studied with reduced injection dose and reconstruction over the entire acquisition time. Our study trained a model on a dataset from one scanner and one tracer, which was not optimal for AI development. Nevertheless, our preliminary results confirmed the potential of our initial hypothesis, albeit in such a challenging cross-scanner and cross-tracer setup. This proof of concept can therefore support the design of more realistic studies in the future, by including a larger and heterogeneous dataset that is not limited by the center, scanner, tracer, disease, or body region. It would be also helpful to further develop algorithms directly based on high-level information extracted from PET raw data. In addition, multimodal methods for dose reduction may be of benefit. Finally, since CT is another major contributor to the total effective dose when performing PET/CT, it would be helpful to investigate deep learning methods for the dose reduction on CT imaging as well. However, this aspect might be more relevant in body PET/CT protocols, where CT is the main contributor to the effective dose whereas the used dose of the radiopharmaceutical is the main contributor to brain PET/CT [62].

## Conclusion

The deep learning approach developed for low-dose PET image enhancement had the potential to be applied on different scanners and tracers with certain limitations. The improvement of image quality by using AI tended to increase with decreasing image quality when applied on cross-scanner and cross-tracer data. When applying high DRFs in cross-tracer applications, potential artifacts must be treated with caution, especially when applied to radiomics feature analysis. Clinical evaluations suggested that using AI is advantageous, although further validation is needed, including in the context of clinical routine. It is reasonable to suggest training with more available data would further consolidate the capability of AI.

## Supplementary Information

ESM 1 (DOCX 2436 kb)
