# Supplementary material for: A cross-scanner and cross-tracer deep learning method for the recovery of standard-dose imaging quality from low-dose PET
Source: Eur J Nucl Med Mol Imaging. 2021 Dec 24;49(6):1843–56. doi: 10.1007/s00259-021-05644-1 (PMC9015984; doi:10.1007/s00259-021-05644-1)
Supplement: Supplementary file 1 — (DOCX 2436 kb) [file 259_2021_5644_MOESM1_ESM.docx]

**SUPPLEMENTARY MATERIAL**

**A cross-scanner and cross-tracer deep learning method for the recovery of standard-dose imaging quality from low-dose PET**

**Deep Neural Network design**

The goal of the generator is to be able to approximate the corresponding full dose image for a given low dose image, while the discriminator aims to distinguish between the synthesized full dose image and the real input. It’s like a competing game between these two: one tries to fool the other with generating full dose image as real as possible, while the other attempts to maximize its capability of not being fooled.

Generator network: The goal of the generator is to be able to approximate the corresponding $I^{Full Dose}$ for a given $I^{Low Dose}$. The network of generator consisting of 16 convolutional layers, which include 8 down-sampling layers for the encoder and 8 up-sampling layers for the decoder. Pooling layers are replaced with stride convolutions to avoid information loss [1].

Discriminator network: The discriminator network aims to distinguish between the $I^{Synthesized Full Dose}$ generated by the generator model, and the real input $I^{Full Dose}$. The discriminator takes either a real full-dose image or a synthesized one as input, and determines whether the input is real or not. The architecture of the discriminator network contains eight convolution blocks and a fully connected block is connected at the end, with sigmoid as activation to output a probability, to determine whether the input dosimetry image is real or synthetic.

We specifically modified our model for cross-scanner and cross-tracer application including the following steps: 1. We designed the generator as a U-net like architecture, which features the skip connection (refers to “Concatenate” in Figure 2). Considering the fact that low-dose images and full-dose images both are renderings of the same underlying structure, a great deal of low-level information is shared between them. The skip connection was designed here to shuttle low-level feature representation from encoding layers directly to decoding layers [2]. 2. We also introduced the batch normalization (BN) [3] to both generator and discriminator, which helps the network to learn more efficiently by normalizing each batch with its mean and standard deviation. Techniques like skip connection and BN allow the network architecture to be much deeper, which endows the network better capability of generalization, and makes it particularly suitable for the task of cross-scanner and cross-tracers PET enhancement. 3. We proposed an extended objective function on top of the original GAN [4] as:

$$\min_{G}\max_{D}V\left( D,G \right)=\sum_{i=1}^{n} \log\left( D_{\theta_{D}}\left( I^{Low Dose}, I^{Full Dose} \right) \right)+log\left( 1-D_{\theta_{D}}\left( {I^{Low Dose},G}_{\theta_{G}}\left( I^{Low Dose} \right) \right) \right)$$

In contrast to conditioning the generation of images on random noise drawn from specific distribution, this objective function takes an input of low-dose PET image. Furthermore, the adversarial loss of our model also included voxel-wise content loss alongside image-wise loss, to ensure spatial alignment of the enhanced full dose images with the ground truth:

$L_{\text{content loss}}=\sum_{i=1}^{n} \left| I^{\text{Enhanced Full Dose}}-I^{\text{Full Dose}} \right|_{2}$

where $I^{Enhanced Full Dose}=G_{\theta_{G}}\left( I^{Low Dose} \right)$

Therefore, the overall objective function was defined as:

$$\min_{G}\max_{D}V\left( D,G \right)+\lambda V\left( G \right)=\sum_{i=1}^{n} \log\left( D_{\theta_{D}}\left( I^{Low Dose}, I^{Full Dose} \right) \right)+log\left( 1-D_{\theta_{D}}\left( {I^{Low Dose},G}_{\theta_{G}}\left( I^{Low Dose} \right) \right) \right)+\lambda\left| G_{\theta_{G}}\left( I^{Low Dose} \right)-I^{Full Dose} \right|_{2}$$

Training details: We employed the Adam solver [5] with a batch size of 4 and a learning rate of 0.0002. In order to facilitate efficient access to this large number of images during training, the dataset was organized into a single data object in HDF5 (Hierarchical Data Format 5). All of our experiments are implemented in TensorFlow and trained on our NVIDIA GeForce GTX 1080 Ti graphic cards.

We trained two versions of the neural networks at first, henceforth referred to as the individual model and the generalized model. For the individual model, the network was trained on a paired set of images at the standard-dose and a given DRF, leading to four individual models (DRF=2,4,10,20). The trained individual models were later tested only on corresponding DRF test datasets. The generalized model was trained by mixing the image pairs of different DRF to explore generalization capability. Afterwards, one trained generalized model, which was developed with data only up to DRF 20, was tested on all test datasets from DRF 2 to 100.

As shown in Supplementary Figure S1, both the individual model and the generalized model were able to achieve an overall better physical performance compared to non-AI enhanced images, on the test group of brain disease with DMI [^18^F]FDG, and the improvement tended to get greater with the increase of DRF, especially for the generalized model. But the generalized model showed overall better performance on cross-scanner or cross-tracer applications. The comparison of the individual and the generalized model demonstrated the effectiveness of data augmentation of GANs or deep learning. The blending of training images from multiple levels of DRF can be viewed as a data augmentation technique, which has already proved useful in other applications [6]. The main limitation of the individual model is its lack of generalizability. In this respect, the generalized model outperforms when enhancing data from different scanners, data from different tracers as well as data of high DRFs (10, 20), and even DRF (50, 100) which were not included in the development. The reason for this could be that the generalized model was trained on overall more data including data with low DRF, which contains more extractable latent structural information, which may thus contribute to better quality of images. However, applying these enhancement methods for different tracers at high DRF may yield unreliable results.

**Evaluation based on Physical Metrics, Clinical and Radiomics features**

**Physical Metrics**

To evaluate the quality of the enhanced images, we calculated and compared the following metrics: 1. Normalized root mean squared error (NRMSE); 2. Peak signal-to-noise ratio (PSNR); 3. Structural similarity index measurement (SSIM) [7]. The NRMSE is defined as:

$$NRMSE=\frac{\sqrt{\sum_{i=1}^{n} \frac{\left( y_{true}-y_{pred} \right)^{2}}{n}}}{max\left( y_{true})-min(y_{pred} \right)}$$

where $y_{\mathrm{true}}$ is the Full dose image and $y_{pred}$ is the enhanced PET image, and it measures the overall pixel-wise intensity deviation between these two. The PSNR is defined as:

$$PSNR=10\log_{10} \left( \frac{VR^{2}}{\left\| y_{true}-y_{pred} \right\|_{2}^{2}} \right)$$

where V is the total amounts of voxels and R represents the range of the intensity of the Full dose image, and $\left\| y_{true}-y_{pred} \right\|_{2}^{2}$ computes the mean squared error between it and the enhanced PET image. The pixel-wise quantities are easily calculated and compared and have straightforward interpretations. However, they do not correspond well with the sort of errors that humans perceive, particularly blurring and smearing artifacts, and images with identical NRMSE values may appear substantially different [7]. Additional measures that more accurately reflect perceived image quality are therefore desirable.

$$\mathrm{SSIM} \left( x,y \right)=\frac{\left( 2\mu_{x}\mu_{y}+C_{1} \right)\left( 2\sigma_{xy}+C_{2} \right)}{\left( \mu_{x}^{2}+\mu_{y}^{2}+C_{1} \right)\left( \sigma_{x}^{2}+\sigma_{y}^{2}+C_{2} \right)}$$

where $\mu_{x}$, $\mu_{y}$ are the averages of images X and Y, and $\sigma_{x}$, $\sigma_{y}$ are their standard deviations, respectively. $C_{1}$ and $C_{2}$ are two positive constants to avoid a null denominator. Theoretically, image with lower NRMSE, higher PSNR and SSIM closer to 1 represent higher synthesis quality.

**Clinical and Radiomics features**

A combination of manual and threshold-based methods was applied to delineate and segment tumor lesions on standard full dose images. This was followed by standardized uptake value (SUV) discretization as well as statistical analysis. Clinical parameters and radiomics features were both included for the analysis, and we selected the mostly applied features based on references. As a result, SUV_max_, SUV_mean_ were selected for the analysis of both [^18^F]FDG images and [^18^F]FET images [8-12]. Clinical features including total lesion glycolysis (TLG), and radiomics features such as Root Mean Squared, 90Percentile, Median, as well as Joint Average from Gray-level co-occurrence matrix (GLCM) were selected for [^18^F]FDG images [13-15]. For the [^18^F]FET images, radiomics features including Kurtosis from first order features, Gray Level Non-Uniformity and High Gray Level Run Emphasis from Gray-level run-length matrix, Zone Percentage from Gray-level size zone matrix (GLSZM) were selected [16, 17]. More detailed information related to clinical-relevant feature selection can be found in Supplementary Table 2.


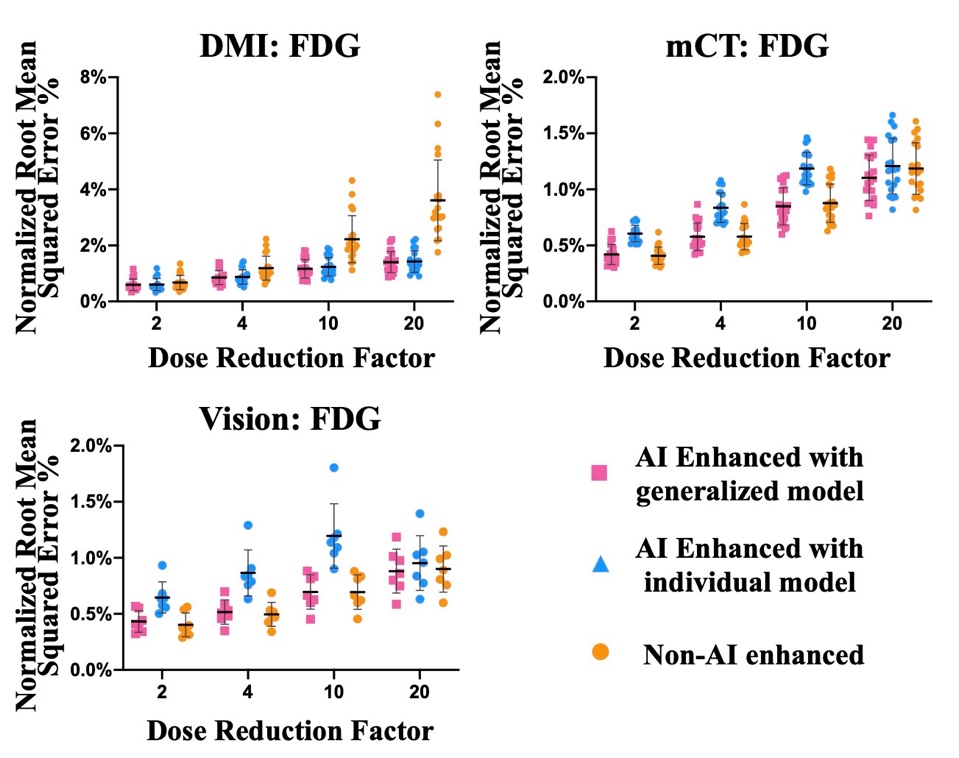


**Supplementary Figure S1.** Figures demonstrate the improvement with the help of artificial intelligence (AI) enhancement in terms of Normalized Root Mean Squared error (NRMSE) on [^18^F]FDG data. Both the individual model and the generalized model were able to achieve an overall better physical performance compared to non-AI enhanced images, on the test group of brain disease with DMI [^18^F]FDG scans, and the improvement tended to get greater with the increase of DRF, especially for the generalized model. Also, the generalized model showed overall better performance on cross-scanner or cross-tracer applications.


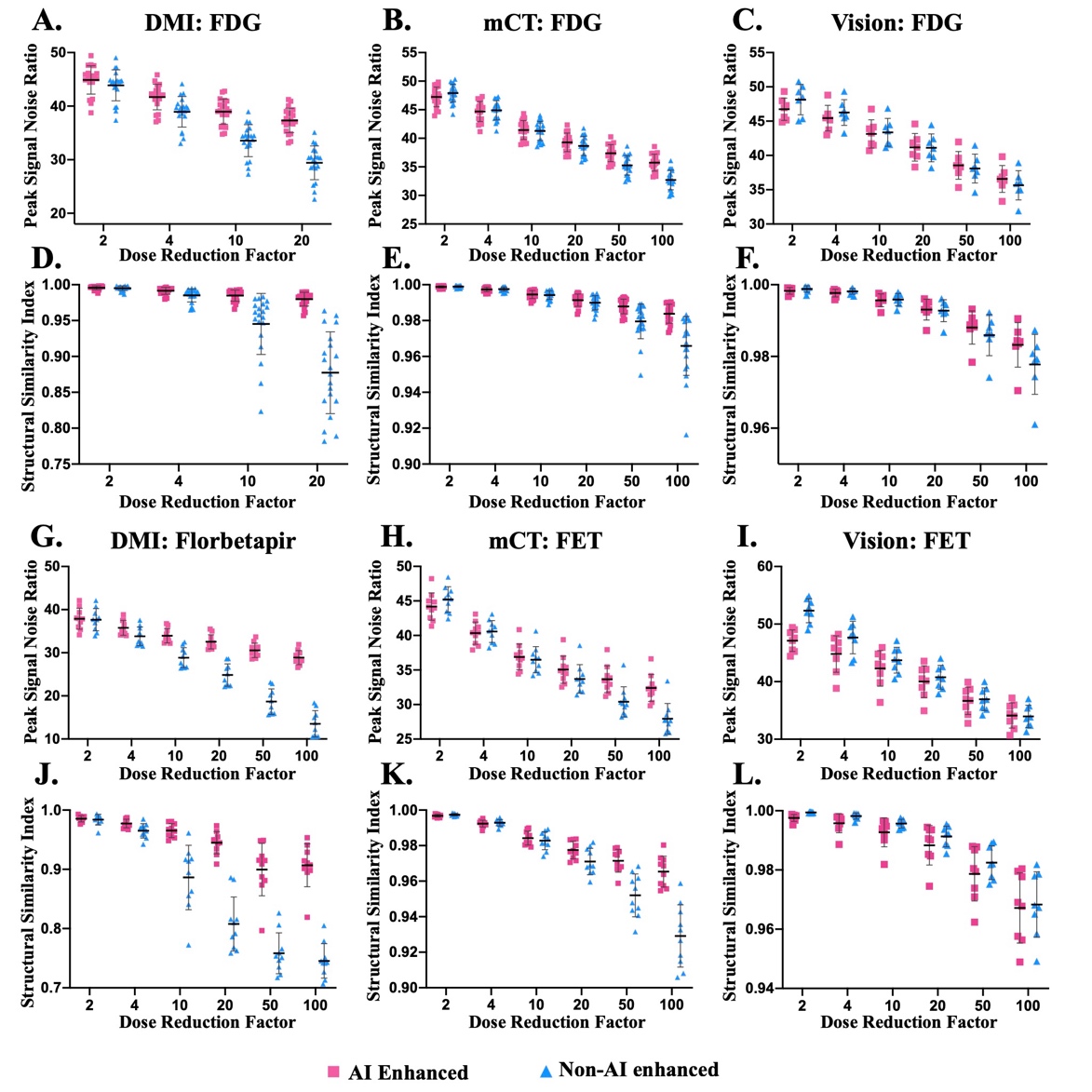


**Supplementary Figure S2.** Improvement with the help of the developed artificial intelligence (AI) enhancement in terms of PSNR (peak signal-to-noise ratio) and SSIM (structural similarity index measurement) in a cross-scanner and cross-tracer setting. A-F): Comparison of PSNR and SSIM between images with and without AI enhancement at different dose reduction factors (DRFs) for the [^18^F]FDG data from DMI (A: GE, Discovery MI), mCT (B: Siemens, Biograph mCT) and Vision (C: Siemens, Biograph Vision), respectively. G-L): Comparison of PSNR and SSIM between images with and without AI enhancement at different dose reduction factors (DRFs) for the [^18^F]Florbetapir data from DMI (A: GE, Discovery MI), [^18^F]FET from mCT (B: Siemens, Biograph mCT) and Vision (C: Siemens, Biograph Vision), respectively.


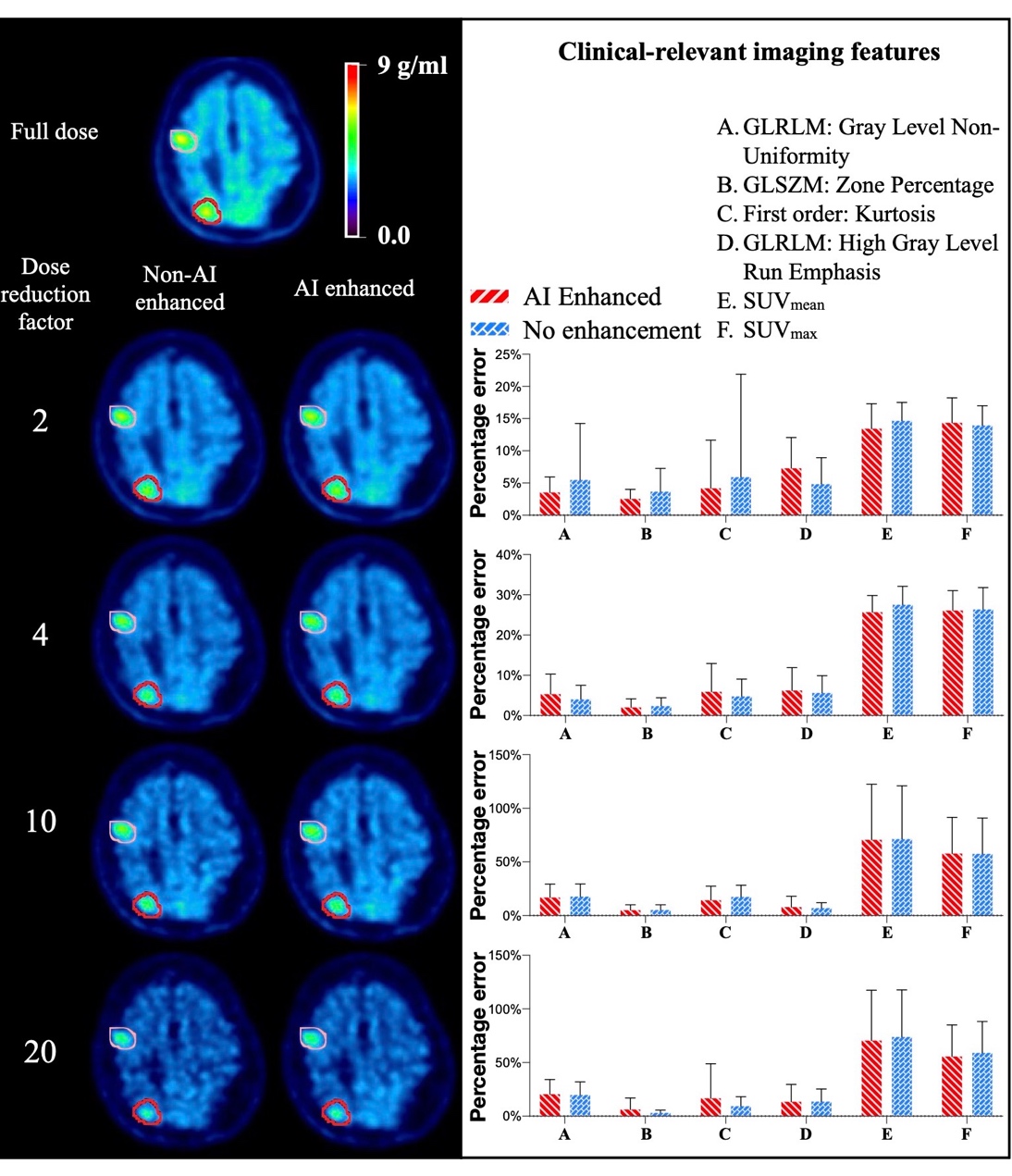


**Supplementary Figure S3.** Clinical-relevant imaging features analysis — mCT (Siemens, Biograph mCT): [^18^F]FET. Results show that none of the clinical features of the [^18^F]FET image benefited from the enhancement.


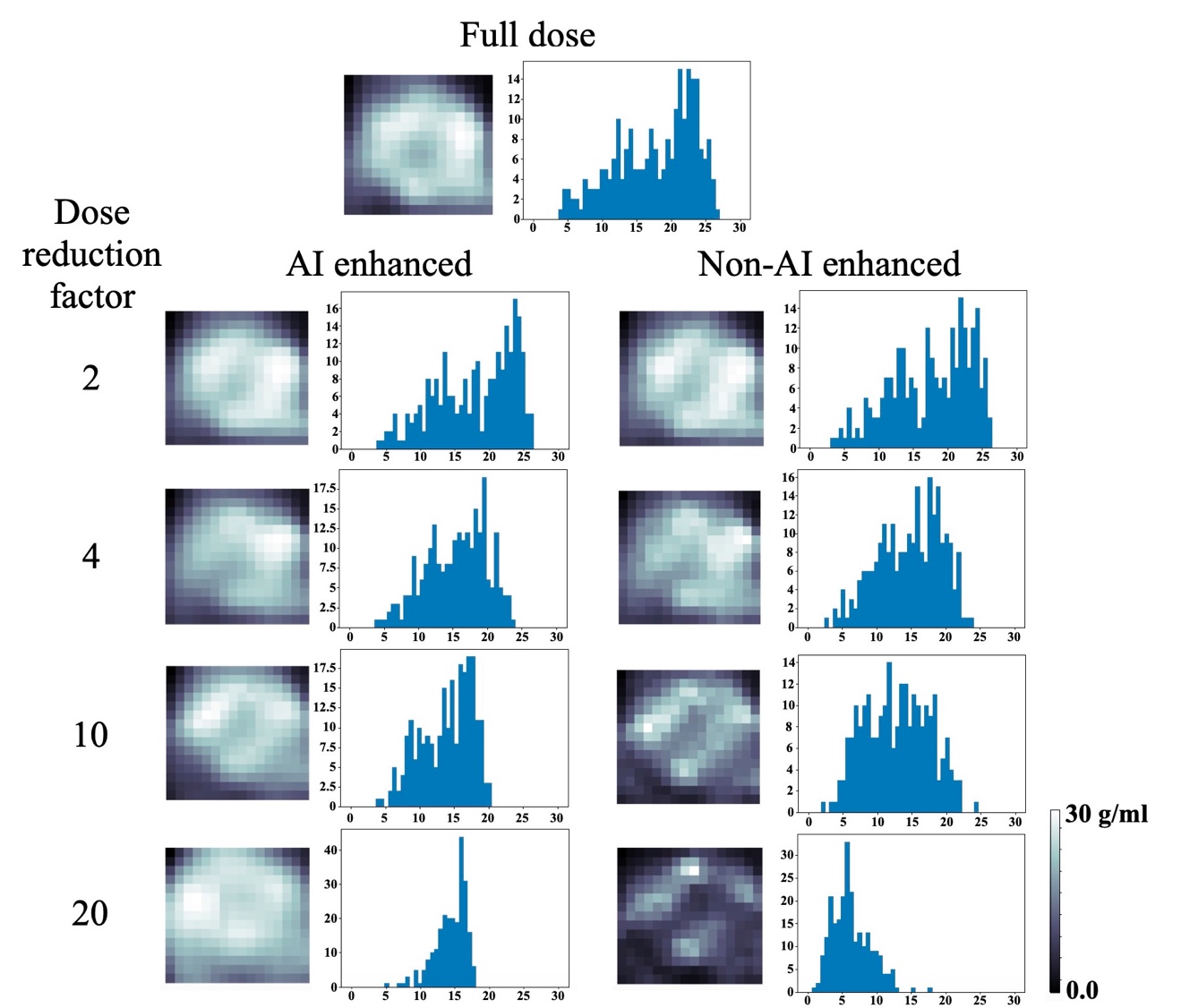


**Supplementary Figure S4.** Cropped lesions examples and corresponding intensity histograms of [^18^F]FDG data. The x-axis of the histogram represents the standardized uptake value (SUV), and the y-axis of shows the counts of voxels which fell into a certain range of the SUV. Histogram results showed AI enhanced images were more similar to the full-dose images, compared to non-AI enhanced images on [^18^F]FDG dataset.
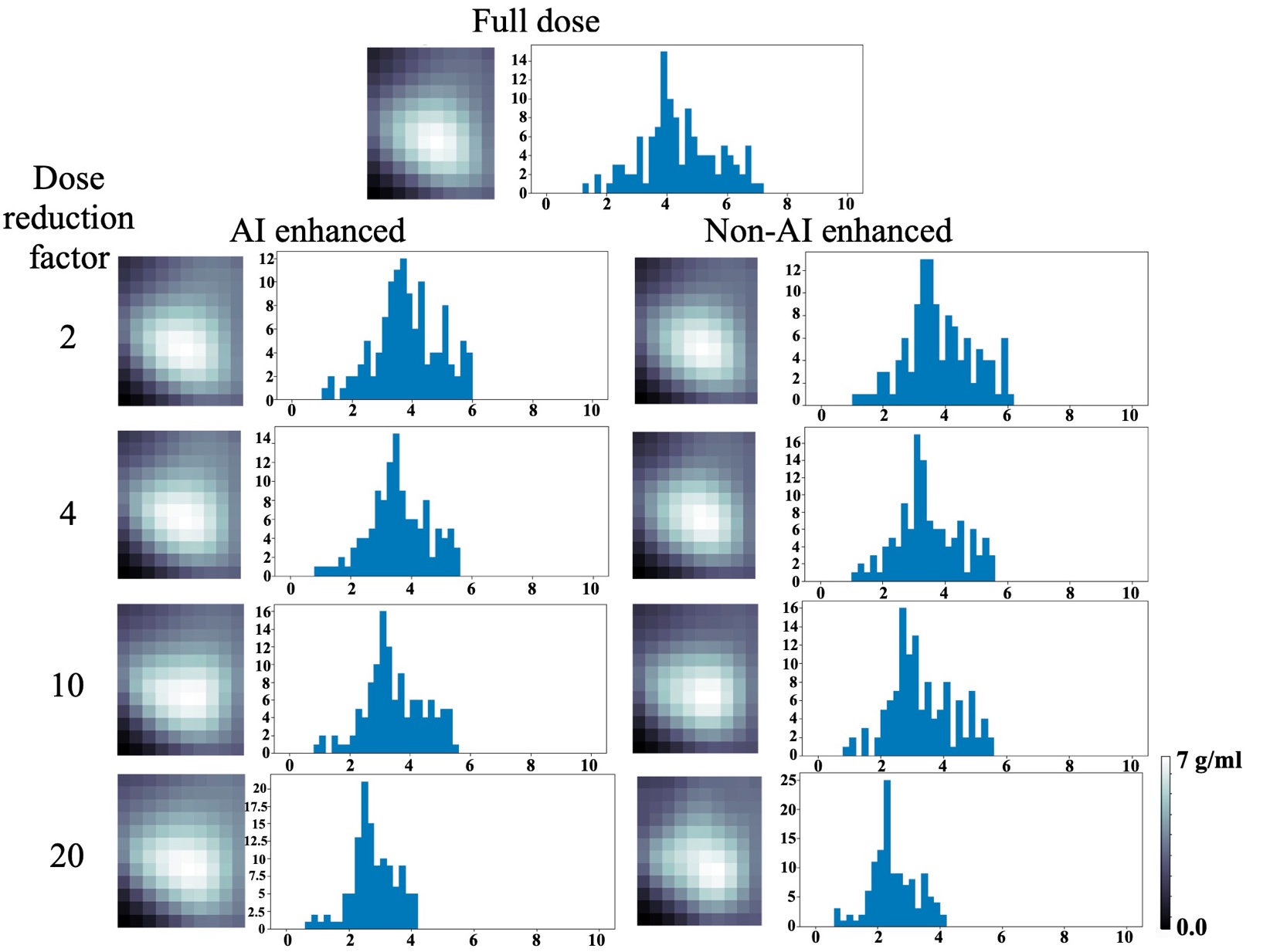


**Supplementary Figure S5.** Cropped lesions examples and corresponding intensity histogram of [^18^F]FET data. The x-axis of the histogram represents the standardized uptake value (SUV), and the y-axis of the counts of voxels which fell into a certain range of the SUV. Histogram results showed AI enhanced images had similar appearance with non-AI enhanced images on [^18^F]FET dataset.


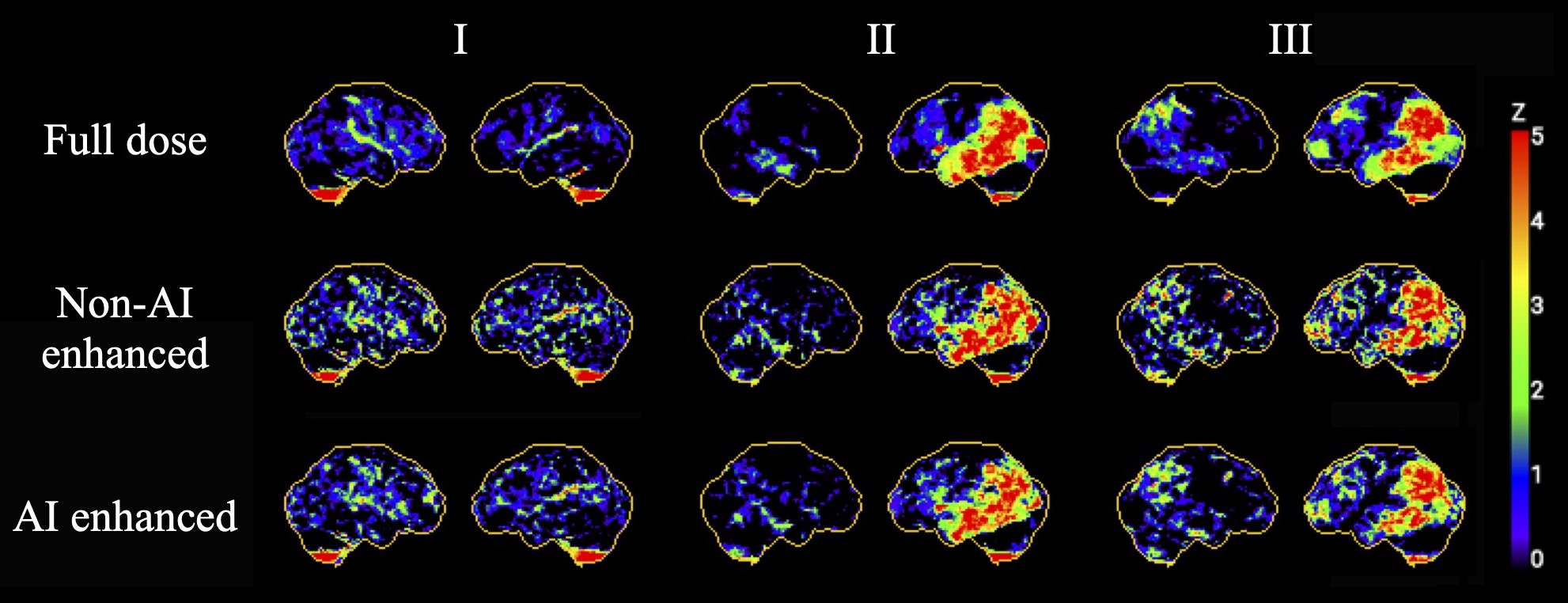


**Supplementary Figure S6.** Exemplary images of 3D-SSP results of three patients (I, II, III) with global cortex as reference region for full dose, DRF=50 non-AI enhanced and DRF=50 AI enhanced images from mCT. Patients referred for further clarification of various neurodegenerative diseases.


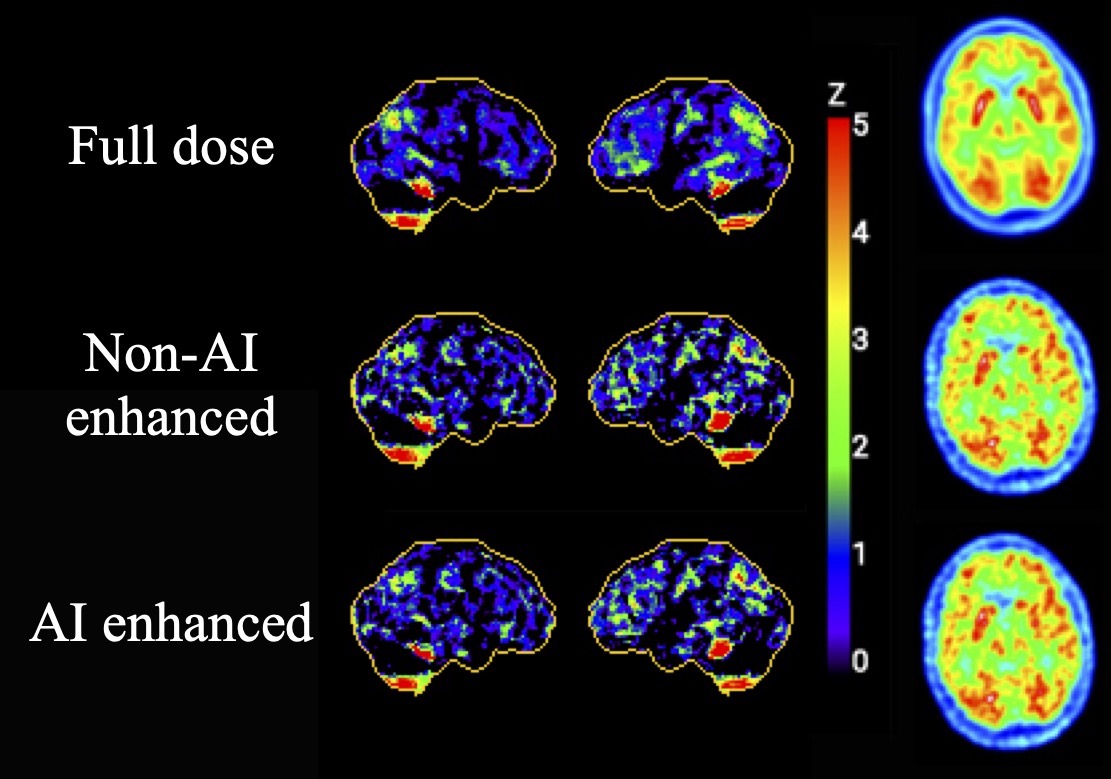


**Supplementary Figure S7.** Exemplary images of 3D-SSP results with global cortex as reference region and corresponding axial slides for full dose, DRF=50 non-AI enhanced and DRF=50 AI enhanced images from Vision. Male patient, 75 years old, referred with depression, listlessness, deficits of executive functions and concentration deficits. Suspicion of neurodegenerative disease especially frontotemporal lobar degeneration.


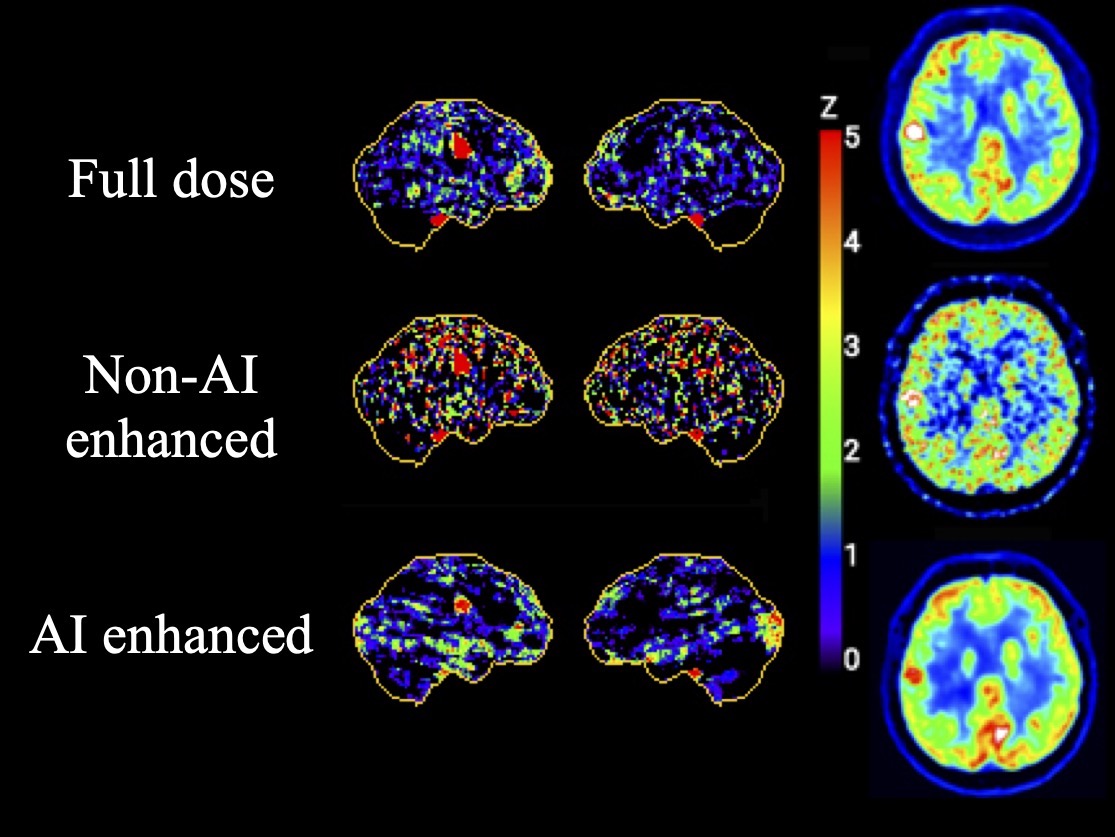


**Supplementary Figure S8.** Exemplary images of 3D-SSP results with global cortex as reference region and corresponding axial slides for full dose, DRF=20 non-AI enhanced and DRF=20 AI enhanced images from DMI (GE, Discovery MI). The hypermetabolic area on the right at the border between frontal and parietal lobes can be seen in the 3D-SSP images of all enhancements even though the quality of the DRF=20 image without AI enhancement is much worse than in the other images. However, in the axial images, the hypermetabolic area in the right hemisphere is barely distinguishable from the adjacent brain areas on the DRF=20 images anymore.


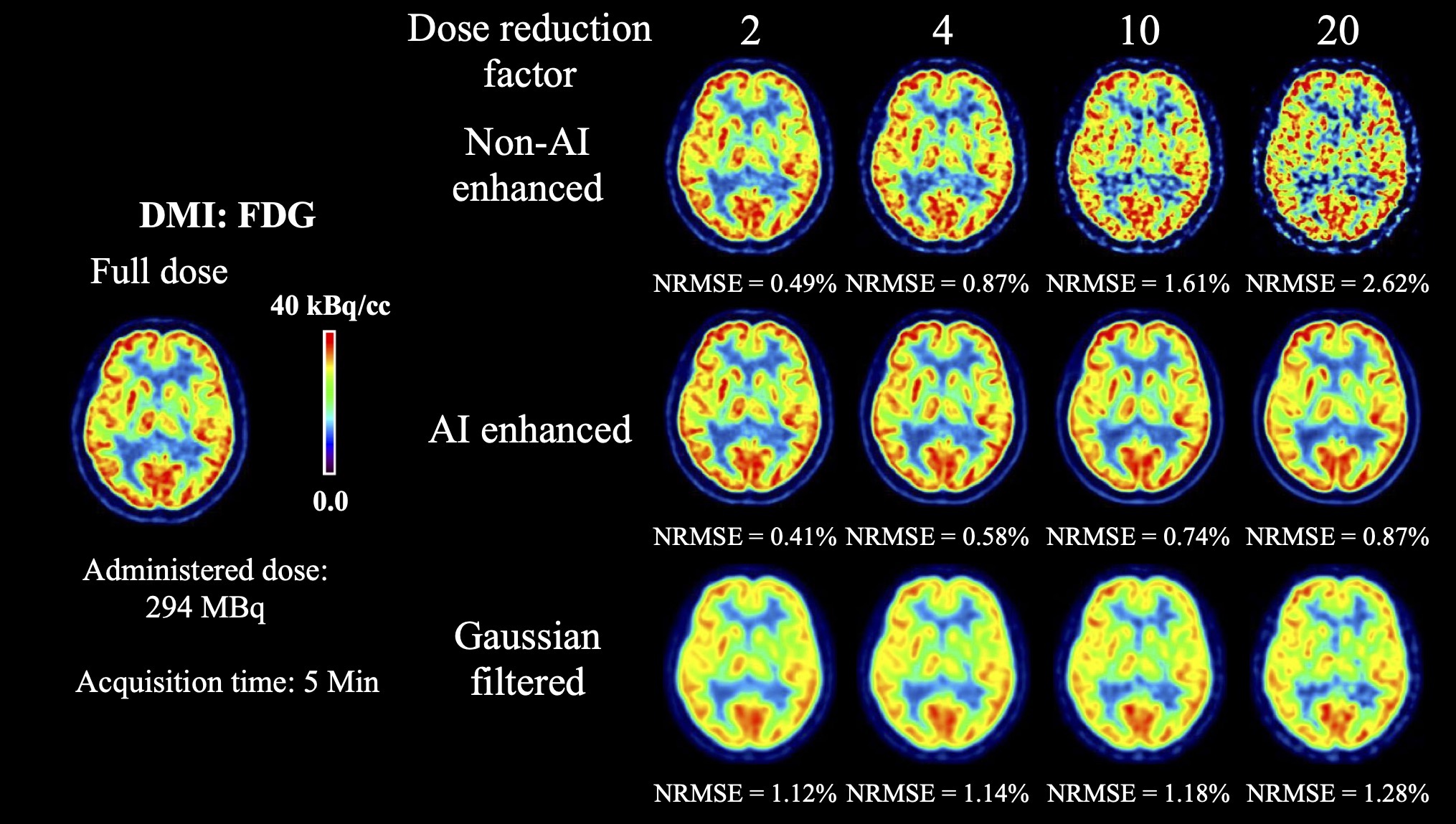


**Supplementary Figure S9.** Example of comparison of AI enhancement and Gaussian filterin of [^18^F]FDG imaging from DMI (GE Discovery MI), the full width at half maximum (FWHM) of Gaussian filtering was set to 6 mm. The advantage of AI enhancement has visually better spatial resolution and contrast.

|  |  | DMI | mCT | Vision |
| --- | --- | --- | --- | --- |
| Scanner properties | Sensitivity | 14 cps/kBq | 9.6 cps/kBq | 16.4 cps/kBq |
|  | Spatial resolution (FWHM, 1cm, radial) | 4.10 mm | 4.3 mm | 3.6 mm |
|  | Scatter fraction | 40.6% | 33.5% | 38.7% |
| Reconstruction parameters | Reconstruction algorithm | OSEM (2 iterations, 17 subsets, 6.4-mm post-filter cutoff) +TOF +PSF +BPL | OSEM (6 iterations, 21 subsets) + TOF + 3D Gaussian 5mm FWHM | OSEM (6 iterations, 5 subsets) + TOF + 3D Gaussian 5mm FWHM |
|  | Matrix size | 256*256 | 256*256 | 256*256 |
|  | Zoom factor | None | 2 | 2 |
|  | Gaussian filter | None | 5 mm | 5 mm |

**Supplementary Table S1.** Scanners properties

|  | **Tracer** | | | | | **Other** | |
| --- | --- | --- | --- | --- | --- | --- | --- |
|  | **[^18^F]FDG** | | | **[^18^F]FET** | |  | |
| **Reference** | Li L, et al. 2019 | Kong Z, et al. 2019 | Wu Y, et al. 2019 | Lohmann P, et al. 2018 | Lohmann P, et al. 2017 | Hatt, M, et al. 2017 | Kim BH, et al. 2015 |
| **Clinical feature** |  |  |  |  | - SUV_max_ | - SUV_max_ - SUV_mean_ | - Metabolic tumor volume (MTV) - Total lesion glycolysis (TLG) |
| **Firstorder** | - Skewness - Root Mean Squared Mean | - Skewness - 90 Percentile - Median | - Skewness - Variance - Entropy - Kurtosis |  | - Skewness - Kurtosis |  |  |
| **Shape** | - Sphericity | - Maximum 2D Diameter Slice |  | - Volume |  |  |  |
| **GLCM** | - Inverse Variance - Imc1 - Autocorrelation | - Joint Average |  |  | - Homogeneity |  |  |
| **GLDM** | - Dependence Non-Uniformity Normalized |  |  |  |  |  |  |
| **GLDM** | - Dependence Non-Uniformity Normalized |  |  |  |  |  |  |
| **GLRLM** | - Run Length Non-Uniformity Normalized - Short Run Low Gray Level Emphasis |  | - Long Run High Gray Level Emphasis | - Run Length Non-Uniformity | - Short Run High Gray Level Emphasis - High Gray Level Run Emphasis - Long Run Emphasis |  |  |
| **GLSZM** |  |  | - Low Gray Level Zone Emphasis - large zone high-gray-level emphasis - Gray Level Variance | - Gray Level Non-Uniformity - Long-zone high grey-level emphasis |  |  |  |
| **NGTDM** |  |  | - Strength - Coasenes |  |  |  |  |

**Supplementary Table S2.** Clinical-relevant imaging features selection reference

REFERENCES

1. Springenberg JT, Dosovitskiy A, Brox T, Riedmiller M. Striving for simplicity: The all convolutional net. arXiv preprint arXiv:14126806. 2014.

2. Ronneberger O, Fischer P, Brox T. U-net: Convolutional networks for biomedical image segmentation. International Conference on Medical image computing and computer-assisted intervention: Springer; 2015. p. 234-41.

3. Ioffe S, Szegedy C. Batch normalization: Accelerating deep network training by reducing internal covariate shift. arXiv preprint arXiv:150203167. 2015.

4. Goodfellow I, Pouget-Abadie J, Mirza M, Xu B, Warde-Farley D, Ozair S, et al. Generative adversarial nets. Advances in neural information processing systems; 2014. p. 2672-80.

5. Kingma DP, Ba J. Adam: A method for stochastic optimization. arXiv preprint arXiv:14126980. 2014.

6. Ren W, Yan S, Shan Y, Dang Q, Sun G. Deep image: scaling up image recognition. arXiv preprint arXiv:150102876. 2015;7.

7. Wang Z, Bovik AC, Sheikh HR, Simoncelli EP. Image quality assessment: from error visibility to structural similarity. IEEE transactions on image processing. 2004;13:600-12.

8. Brooks FJ, Grigsby PWJJoNM. The effect of small tumor volumes on studies of intratumoral heterogeneity of tracer uptake. 2014;55:37-42.

9. Hatt M, Majdoub M, Vallières M, Tixier F, Le Rest CC, Groheux D, et al. 18F-FDG PET uptake characterization through texture analysis: investigating the complementary nature of heterogeneity and functional tumor volume in a multi–cancer site patient cohort. 2015;56:38-44.

10. Hatt M, Tixier F, Pierce L, Kinahan PE, Le Rest CC, Visvikis DJEjonm, et al. Characterization of PET/CT images using texture analysis: the past, the present… any future? 2017;44:151-65.

11. Presotto L, Bettinardi V, De Bernardi E, Belli M, Cattaneo G, Broggi S, et al. PET textural features stability and pattern discrimination power for radiomics analysis: An “ad-hoc” phantoms study. 2018;50:66-74.

12. Kim BH, Kim S-J, Kim K, Kim H, Kim SJ, Kim WJ, et al. High metabolic tumor volume and total lesion glycolysis are associated with lateral lymph node metastasis in patients with incidentally detected thyroid carcinoma. 2015;29:721-9.

13. Kong Z, Lin Y, Jiang C, Li L, Liu Z, Wang Y, et al. 18 F-FDG-PET-based Radiomics signature predicts MGMT promoter methylation status in primary diffuse glioma. 2019;19:58.

14. Li L, Mu W, LIU Z, Liu Z, Wang Y, Ma W, et al. A Non-invasive Radiomic Method Us 18F-FDG PET Predicts Isocitrate Dehydrogenase Genotype and Prognosis in Patients with Glioma. 2019;9:1183.

15. Wu Y, Jiang J-H, Chen L, Lu J-Y, Ge J-J, Liu F-T, et al. Use of radiomic features and support vector machine to distinguish Parkinson’s disease cases from normal controls. 2019;7.

16. Lohmann P, Kocher M, Ceccon G, Bauer EK, Stoffels G, Viswanathan S, et al. Combined FET PET/MRI radiomics differentiates radiation injury from recurrent brain metastasis. 2018;20:537-42.

17. Lohmann P, Stoffels G, Ceccon G, Rapp M, Sabel M, Filss CP, et al. Radiation injury vs. recurrent brain metastasis: combining textural feature radiomics analysis and standard parameters may increase 18 F-FET PET accuracy without dynamic scans. 2017;27:2916-27.
